# Computational inference of the structure and regulation of the lignin pathway in *Panicum virgatum*

**DOI:** 10.1186/s13068-015-0334-8

**Published:** 2015-09-17

**Authors:** Mojdeh Faraji, Luis L. Fonseca, Luis Escamilla-Treviño, Richard A. Dixon, Eberhard O. Voit

**Affiliations:** The Wallace H. Coulter Department of Biomedical Engineering, Georgia Institute of Technology and Emory University, 313, Ferst Drive, Atlanta, GA 30332 USA; BioEnergy Sciences Center (BESC), Oak Ridge National Lab, Oak Ridge, TN USA; Department of Biological Sciences, University of North Texas, 1155 Union Circle #305220, Denton, TX 76203-5017 USA

**Keywords:** Biochemical systems theory, Lignin biosynthesis, *Panicum virgatum*, Pathway analysis, Recalcitrance, Switchgrass

## Abstract

**Background:**

Switchgrass is a prime target for biofuel production from inedible plant parts and has been the subject of numerous investigations in recent years. Yet, one of the main obstacles to effective biofuel production remains to be the major problem of recalcitrance. Recalcitrance emerges in part from the 3-D structure of lignin as a polymer in the secondary cell wall. Lignin limits accessibility of the sugars in the cellulose and hemicellulose polymers to enzymes and ultimately decreases ethanol yield. Monolignols, the building blocks of lignin polymers, are synthesized in the cytosol and translocated to the plant cell wall, where they undergo polymerization. The biosynthetic pathway leading to monolignols in switchgrass is not completely known, and difficulties associated with in vivo measurements of these intermediates pose a challenge for a true understanding of the functioning of the pathway.

**Results:**

In this study, a systems biological modeling approach is used to address this challenge and to elucidate the structure and regulation of the lignin pathway through a computational characterization of alternate candidate topologies. The analysis is based on experimental data characterizing stem and tiller tissue of four transgenic lines (knock-downs of genes coding for key enzymes in the pathway) as well as wild-type switchgrass plants. These data consist of the observed content and composition of monolignols. The possibility of a G-lignin specific metabolic channel associated with the production and degradation of coniferaldehyde is examined, and the results support previous findings from another plant species. The computational analysis suggests regulatory mechanisms of product inhibition and enzyme competition, which are well known in biochemistry, but so far had not been reported in switchgrass. By including these mechanisms, the pathway model is able to represent all observations.

**Conclusions:**

The results show that the presence of the coniferaldehyde channel is necessary and that product inhibition and competition over cinnamoyl-CoA-reductase (CCR1) are essential for matching the model to observed increases in H-lignin levels in 4-coumarate:CoA-ligase (4CL) knockdowns. Moreover, competition for 4-coumarate:CoA-ligase (4CL) is essential for matching the model to observed increases in the pathway metabolites in caffeic acid *O*-methyltransferase (COMT) knockdowns. As far as possible, the model was validated with independent data.

**Electronic supplementary material:**

The online version of this article (doi:10.1186/s13068-015-0334-8) contains supplementary material, which is available to authorized users.

## Background

About 440 million years ago plants started to leave the oceans and inhabit land [1, 2]. The emergence of lignin during this time was an adaptation to the new environment and, specifically, a response to gravity and to limitations in accessing water. The new life also demanded plants to store water and develop systems of water transfer. The plant furthermore needed to grow in height in order to have enough access to sunlight and oxygen. Plants ultimately accomplished these multiple tasks through their xylem structures, of which lignin is a key constituent. Lignin is a phenolic polymer that is woven around and between cellulose and hemicellulose within the secondary cell wall; it provides strength and facilitates water transfer in plants. A consequence of these significant benefits for plants is that lignin is very difficult to decompose, because it is an irregular polymer that contains aromatic rings. This resistance against decomposition and digestion is known as recalcitrance. It is arguably the most important barrier to industrializing second-generation biofuels, and in particular the production of ethanol from inedible plant parts as sustainable and affordable biofuels, because recalcitrance necessitates additional treatment steps, such as hot acid or ammonia baths, to loosen the lignin structure [3–5]. These steps require time and expense and therefore reduce feasibility and cost effectiveness. Moreover, most of the pretreatments are not environmentally friendly [6, 7]. Outside the biofuel industry, recalcitrance affects forage digestibility, and progress toward reducing recalcitrance could have a significant impact on the cattle and sheep industry [8].

Numerous attempts have been made in recent times to manipulate the lignin content and composition in candidate plants for biofuel production. Many of these studies relied on the assumption that the lignin biosynthesis pathway was known. However, this is not necessarily the case, especially in understudied plant species, and the precise pathway structure is often unclear and requires dedicated research for such species. For instance, *Selaginella moellendorffi* and *Medicago truncatula* have basically similar lignin pathways, which however differ in some of their metabolic branch points as well as their enzyme properties [9–11]. Beyond the topological structure, it is not surprising that different species have evolved distinct regulatory control patterns. The immediate consequence of such discrepancies for the biofuel industry is that the direct extrapolation of knowledge, methods and treatments from one species to another is not necessarily valid. Moreover, it is well known that pathway systems are highly nonlinear and difficult to predict with intuition alone. A feasible strategy is therefore to employ computational approaches of systems biology and metabolic engineering.

The design of suitable models for this purpose is not trivial. First, it is generically unclear which mathematical representations are optimal for describing a natural system. Second, one cannot be sure that information or data from one species can be assumed to be valid in another species, even if the two are closely related. Similarly, it has been shown many times that data obtained in vitro are not necessarily applicable in vivo [10–14]. At the same time, species-specific experiments are time consuming and expensive. Mechanistic models based on enzyme kinetics seem to be an intriguing choice, but it has been shown that mechanistic models are not always good solutions, for instance, if parameter values and enzymatic rate laws are based on strong assumptions like bulk reactivity that are not necessarily satisfied in vivo [12]. An alternative that was recently proposed is the characterization of in vivo-like kinetics [13], which however is costly and time consuming and would still require extensive validation, which however is seldom truly achieved [12]. An additional challenge for the design of models is the scarcity and quality of test and validation data, which pose a significant obstacle to all analyses of relatively understudied species.

In this study we analyze the lignin biosynthesis pathway in switchgrass, *Panicum virgatum*, with computational means of systems biology. The analysis is based on a dataset from stem and tiller tissue that consists of the lignin content (H, G and S lignin) and the S/G lignin ratio in wild type and in four transgenic lines (4CL, CCR1, CAD and COMT knockdowns). To some degree, details of the in vitro kinetics of some of the pathway enzymes have also been determined by one of our labs. Our approach here is to develop computational models that characterize the structure and regulatory control patterns of lignin biosynthesis in *P. virgatum* at a systemic level. The goals of this modeling approach are, first, to explain the experimental results from wild type and transgenic lines and, second, to devise a rational basis for strategies to manipulate the pathway toward reduced recalcitrance.

## Results

The results are described in a sequence that follows our step-by-step model design and conveys our rationale for utilizing the observations to remediate discrepancies with the data and for suggesting the investigation of new features to the model in the next step of the analysis. We begin by assessing the pathway structure in switchgrass as it is alleged in the current literature. Next, we examine possible channeling of CCR/CAD, which has been reported for the lignin pathway in alfalfa [5, 14], but not in switchgrass. Even accounting for the possibility of channeling, the experimental data regarding H lignin cannot be captured at this point. Thus, we investigate the effects of product inhibition and competitive inhibition. In the next phase, 4CL inhibition is added as a potential explanation for the accumulation of 4CL substrates, along with a simultaneous decrease in coniferaldehyde in the COMT knockdown. Finally, principal component analysis is performed to investigate the distribution of parameters within the high-dimensional parameter space and to reduce the feasible subspace of parameter values. The results section ends with a validation of the model.

### Reaction system of lignin biosynthesis in switchgrass

**Fig. 1:**
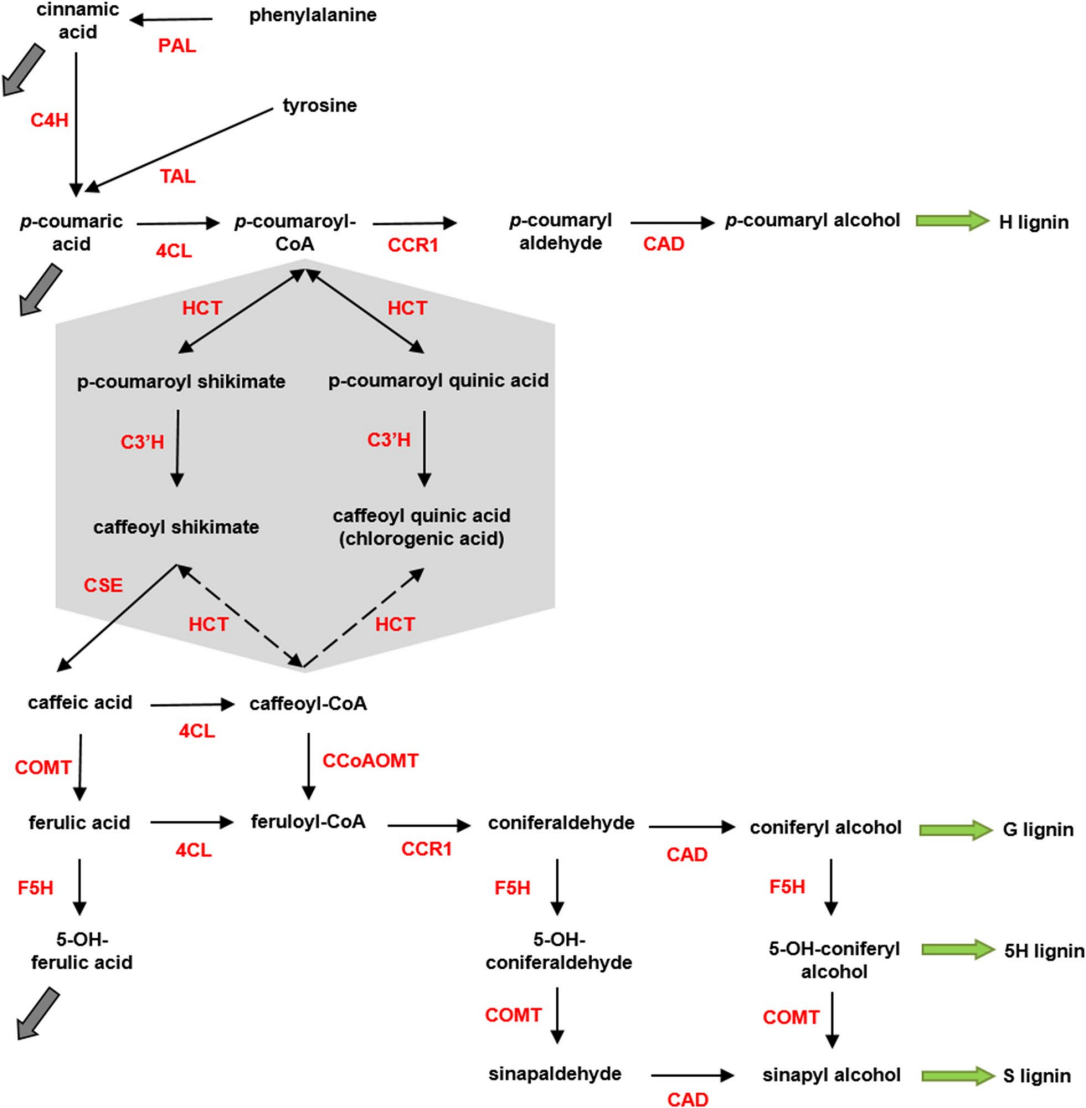
Lignin biosynthesis pathway. *Dashed arrows* represent the traditionally accepted pathway of lignin biosynthesis, while the *arrow* from caffeoyl shikimate to caffeic acid captures a newly discovered enzymatic activity [39] now known to be present in switchgrass. Caffeoyl shikimate esterase turns caffeoyl shikimate into caffeic acid and circumvents the previously accepted route. 4CL has recently been shown to exhibit activity towards caffeic acid and ferulic acid in switchgrass by which a new network topology is introduced for switchgrass lignin biosynthesis. Note that tyrosine is shown here, but not included in the model

The traditionally accepted lignin biosynthesis pathway branches at *p*-coumaroyl CoA to provide S and G-lignin precursors (Fig. 1). The hexagon in this figure shows the details of this branch point. It was also previously assumed, based on studies in the dicots *A. thaliana* and *N. benthamiana*, that *p*-coumaroyl CoA is converted to *p*-coumaroyl shikimate and *p*-coumaroyl quinic acid by HCT. Subsequently, both products, *p*-coumaroyl shikimate and *p*-coumaroyl quinic acid, were shown to be converted to caffeoyl shikimate and caffeoyl quinic acid, respectively [15]. The enzyme for these unidirectional reactions is C3′H. Downstream, HCT was proposed to operate in the reverse direction to convert caffeoyl shikimate and caffeoyl quinic acid into caffeoyl-CoA.

**Fig. 2:**
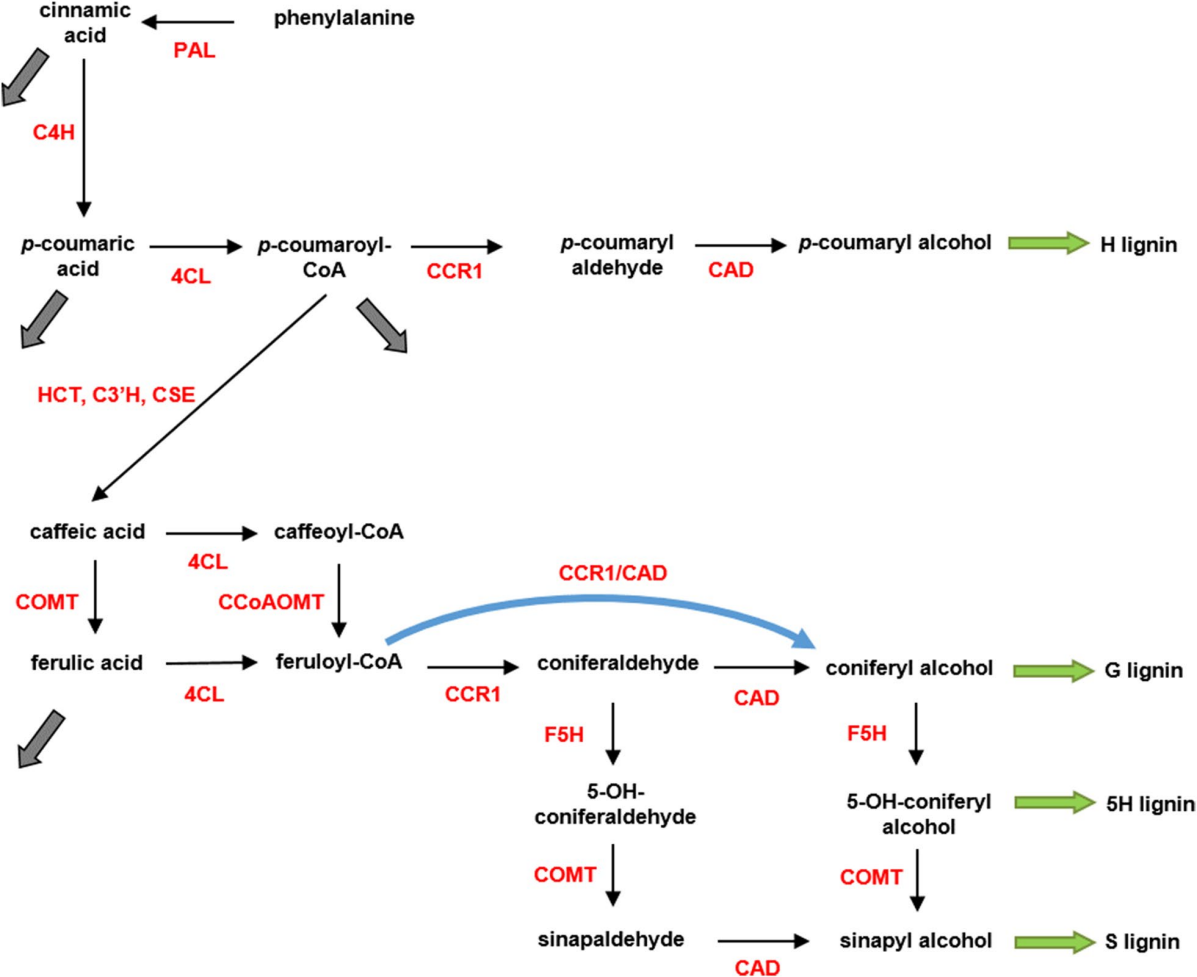
Revised and simplified pathway in switchgrass. By eliminating HCT from the diagram in Fig. 1 and adding CSE, the pathway system becomes simpler. The *right* branch in the *grey box* in Fig. 1 is merged into an efflux and the *left* branch is simplified to a one-step process. It is hypothesized that a specific functional channel could facilitate the conversion of feruloyl-CoA into coniferyl alcohol. Such a channel could be the result of co-localization of the involved pathway enzymes

A recent study demonstrated that this pathway organization is unlikely to occur in switchgrass [16]. Based on kinetic measurements of PvHCT1a, PvHCT2a and PvHCT-Like1, it was shown that caffeoyl shikimate is not converted to caffeoyl-CoA by the reverse HCT reaction, but is more likely converted into caffeic acid through caffeoyl shikimate esterase, and that this step is actually the main route of mass transfer into the pathway towards S and G monolignols. As indicated with dashed arrows in Fig. 1, HCT is not active in the formation of caffeoyl-CoA. This new information helps us reduce the steps in Fig. 1. It has furthermore been suggested that cinnamic acid is a precursor for salicylic acid; this process is represented by the thick grey arrow [5]. Similarly, a considerable portion of ferulic acid leaves the pathway [17]. Finally, the efflux out of *p*-coumaric acid acts to avoid accumulation of the metabolite in the 4CL knockdown strain (Fig. 1). These simplifications yield the pathway diagram in Fig. 2.

At this point, it is not entirely clear whether the lignin pathway in switchgrass contains caffeyl aldehyde. It appears that this is not the case, and the following analysis assumes that caffeyl aldehyde is indeed not produced. Nonetheless, since other species do generate this intermediate, the Additional file 1: Text S1 analyzes this case.

Large-scale simulation studies with this pathway structure lead to irreconcilable differences between the experimental data and the model results, which indicate that the model has genuine flaws. In particular, the dynamics of the different lignin species cannot be explained for the various transgenics (data not shown).

### Channeling

**Fig. 3:**
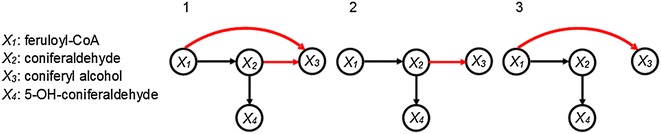
Topological Configurations. Three pathway structures are plausible when a CCR1/CAD channel is considered. Configuration 2 lacks the channel, while the other two configurations represent alternatives involving the channel

Experimental and theoretical work in alfalfa has suggested that functional enzymatic channeling likely occurs at the coniferaldehyde node [5, 14]. According to this suggestion, the “G-channel” facilitates the use of feruloyl-CoA for the production of coniferyl alcohol, which is the precursor of the G monolignol (Fig. 2). We investigate the same channeling hypothesis here as a possibility. Specifically, we use pertinent experimental data from switchgrass to analyze the feasibility of different hypothetical pathway topologies. The potential existence of a functional complex consisting of CCR1/CAD leads to three possible pathway topologies that satisfy the requirement of mass conservation (Fig. 3).

Each of these so-far unregulated topologies was modeled as a generalized mass action (GMA) model, whose parameter values were obtained with a sophisticated large-scale sampling scheme (see “Methods”). Although all topologies were found to be consistent with most of the experimental results, no topology was compatible with the accumulation of H lignin in 4CL knockdown transgenics (Table 1); this situation could not be simulated by any of the candidate models, regardless of the presence or absence of the channel. This strong result suggests the existence of regulatory mechanisms, and considering the structure of the pathway and the branch toward H lignin in particular, we decided to analyze the possible role of product inhibition, which is frequently found in pathway systems in vivo.

**Table 1:** Fold change in lignin monomers, total lignin, and S/G in transgenic plants relative to wild-type plants

	4CL knockdown 40 % [3]	CCR knockdown 50 % [40]	COMT knockdown 30 % [5]	CAD knockdown 30 % [41]
Down-regulation	27–95 %	Up to 75 %	Up to 90 %	55–86 %
H lignin	1.82	NR	NR	NR
G lignin	0.53	~0.75	0.76–0.98	0.67–0.83
S lignin	1.00	~0.75	0.42–0.96	0.58–0.87
Total lignin	0.78	~0.75	0.84–0.96	0.78–0.86
S/G	Increased	Increased	Decreased	Decreased

*NR* not reported

### Product inhibition

**Fig. 4:**
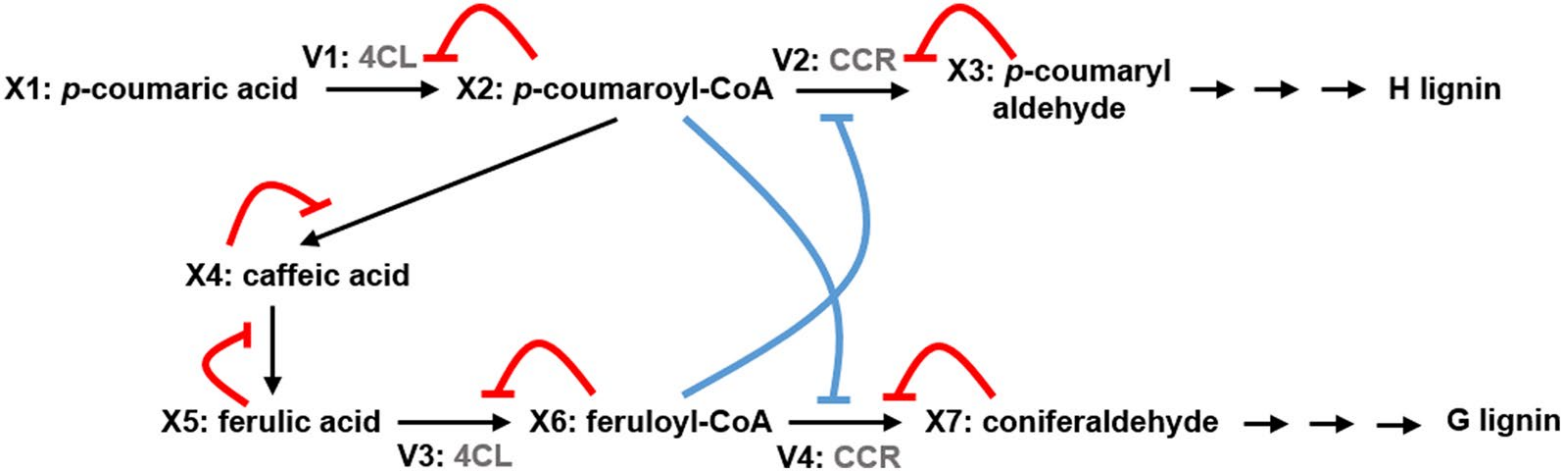
Substrate competition for a shared enzyme, combined with product inhibition. The accumulation of H lignin in the 4CL transgenic line calls for a regulatory mechanism that guides the flow towards the upper branch of the pathway. Direct activation or an inhibited inhibitor can achieve this result. Simulation results support the second option

Experimental results from transgenic plants have demonstrated that H lignin accumulates when the enzyme 4CL is down-regulated [3]. Analyzing this initially counterintuitive observation closer suggests that there might be a wave of accumulation in the metabolites preceding H lignin. Such a wave can be explained with product inhibition (Fig. 4). When an enzyme is down-regulated, the corresponding substrate accumulates. The secondary effect is that the accumulated substrate is by itself a product of a previous reaction whose increased concentration decreases its own rate of production. This backward cascade has an upstream domino effect along the pathway and, depending on the kinetics of the reactions, can lead to the accumulation of upstream metabolites. This observation can be explained by the following chain of events: Down-regulating 4CL leads to a decrease in the products of this enzyme, i.e., *p*-coumaroyl-CoA, caffeoyl-CoA, and feruloyl-CoA. At the same time, product inhibition leads to a backward accumulation in upstream metabolites, which compensates, at least partially, for the initial decrease in *p*-coumaroyl-CoA. Product inhibition is easily incorporated into the GMA model (see “Methods”). Thus, in a new round of simulations, a new set of 100,000 randomly sampled parameter values was generated as before, this time accounting for product inhibition. Again, the configurations satisfying the experimental results were recorded.

Although the simulations showed an improvement regarding the H lignin accumulation in the 4CL knockdown, no topology reached the twofold increase that was reported in the literature [3].

### Substrate competition for shared enzymes

Several enzymes in the lignin pathway catalyze multiple reactions with slightly different substrates, and it is reasonable to assume substrate competition for an enzyme among the multiple substrates. This competition can play an important role in altering the flow of mass in a mutant plant.

We explored the consequences of substrate competition with respect to the pertinent enzyme CCR. The analysis yielded the following result. If CCR favors *p*-coumaroyl-CoA over feruloyl-CoA, due to substrate competition, the flux towards H lignin is increased. In fact, simulation analysis shows that the increase in H lignin is strong enough to match the experimental data.

It could be possible that substrate competition alone would be sufficient for increased H lignin production. We tested this conjecture with a corresponding simulation, which revealed that only the combined model with product inhibition and substrate competition matches the experimental observations. The strength of inhibition is a priori unknown, but simply becomes a parameter value in the GMA model (see “Methods” section). For instance, consider the pathway in Fig. 4, where *X*_2_ and *X*_6_ share the same enzyme for fluxes *V*_2_ and *V*_4_. Blue arrows represent the competition between the substrates, while red arrows represent product inhibition. In this case the equation for *V*_2_ becomes

1 $$V_{2} = \alpha_{2} X_{2}^{{g_{2,2} }} X_{3}^{{ - g_{3,2} }} X_{6}^{{ - g_{6,2} }} Y_{2} ,$$

where *Y*_2_ is the enzyme catalyzing the reaction (CCR).

### Inhibition of 4CL in COMT knockdown transgenics

Although product inhibition and substrate competition improve the consistency between the experimental data and numerical results in CCR1 transgenic plants, the model does not match COMT knockdown data sufficiently well. Specifically, the model does not capture the observed 30 % increase in ferulic acid in COMT knockdowns [4]. This observation becomes even more difficult to explain if one considers the simultaneous 20 % decrease in coniferyl aldehyde. One could speculate that the high accumulation in 5-OH-ferulic acid might trigger a cascade of product inhibition that leads to the accumulation of ferulic acid, but computational results did not support the idea.

Further analysis with the model revealed that the reaction from ferulic acid to feruloyl-CoA, which is catalyzed by 4CL, is the bottleneck. Indeed, the computational results show that this reaction has a flux that is 10 times as large as the efflux from ferulic acid towards 5-OH-ferulic acid. Thus, if the flux towards ferulic acid decreases, any substantial accumulation is impossible unless the 4CL reaction is inhibited. This model-based deduction is indirectly supported by experimental data from one of our labs that exhibit a slight accumulation in the distant *p*-coumaric acid and caffeic acid, which is explained by 4CL inhibition as well (data not shown).

**Fig. 5:**
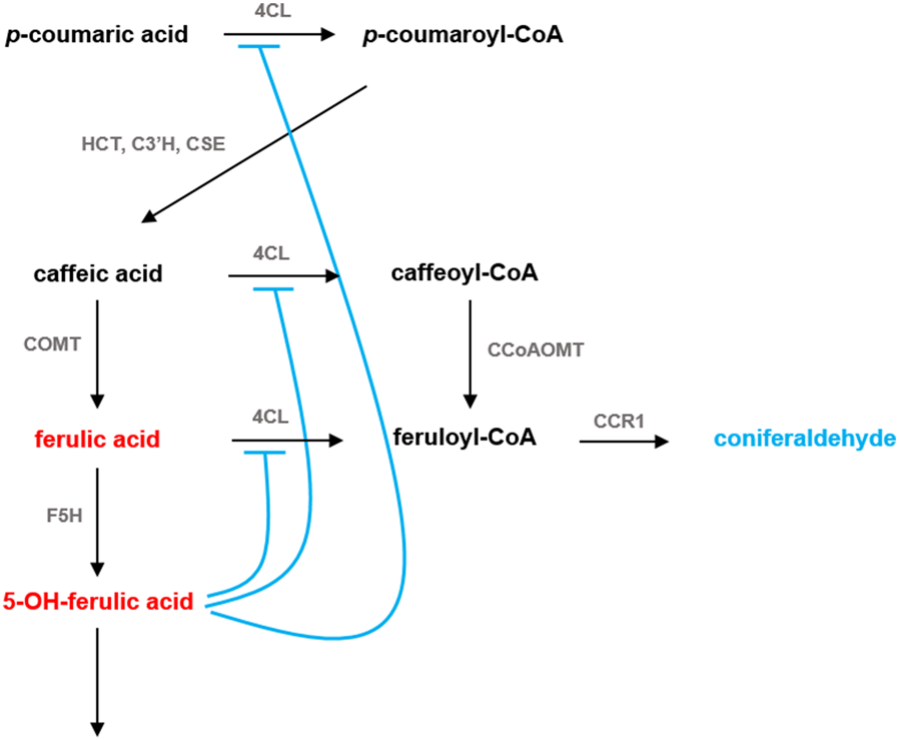
Parallel reactions catalyzed by 4CL. The observed simultaneous accumulation of 4CL substrates and decrease in coniferaldehyde in COMT transgenic lines can be explained with the assumption of an inhibitory effect on the reactions catalyzed by 4CL. 5-OH-ferulic acid could be a candidate for this role. Although 5-OH-ferulic acid is not a substrate for 4CL in switchgrass, it has a similar molecular shape as ferulic acid, so that high concentrations of 5-OH-ferulic acid might exert competitive inhibition that is comparable to the inhibitory effects of ferulic acid

Accounting for the deduced 4CL inhibition in the model leads to simulations that faithfully capture all experimental data associated with the COMT knockdown; in particular, the 4CL substrates accumulate and the concentration of coniferaldehyde decreases, as observed. From a biochemical point of view, one might be interested in identifying the inhibiting agent. As it was mentioned earlier, the 5-OH-ferulic acid concentration increases by 70 % in COMT knockdown plants. While the metabolite has not been identified as a substrate for 4CL, it might be reasonable to assume that it binds to 4CL in high concentrations, due to its molecular similarity, and thereby inhibit the enzyme competitively (Fig. 5). While this hypothesis remains to be experimentally validated, the same type of substrate competition with respect to 4CL has recently been proposed by others [18]. To implement 4CL inhibition in the model in the most generic manner, we simply lowered the corresponding rate constants.

### Compatible configurations

The mathematical model with universal product inhibition, substrate competition for CCR1, inhibition of 4CL, and the possibility of a metabolic channel was subjected to large-scale simulations aimed at inferring the most likely topology of the lignin pathway (recall Fig. 3). Similar to previous simulations, a sample of 100,000 parameter sets was generated to test model consistency with the experimental data and to provide likely kinetic orders for the model (see “Methods”). Intriguingly, the only pathway configuration that is compatible with all available data is Configuration 1 of Fig. 3. Note that the speculated coniferaldehyde channel is indeed present. In fact, no parameter set, using Configurations 2 and 3, could reproduce the experimental data which eliminates the chance to compare the relative performance of the configurations.

### Principal component analysis

To gain a better understanding of the parameter space of the system, principal component analysis (PCA) was performed on the parameter sets that had been filtered by the model criteria. Once the principal components of the parameter space were identified, a new round of simulations was executed. Specifically, a sample of 100,000 parameter sets was generated along the principal directions and within the reduced space. The set was then transformed back to the original coordinates. The successful parameter sets were recorded and are depicted in Additional file 2: Figure S8. Ultimately, principal components 1 through 4 collectively account for 88 % of the variance.

### Model uniqueness

It is theoretically impossible to proof the uniqueness of a model for such complex nonlinear problem, because it is always possible to evoke additional processes in such a fashion that the original model could be subsumed as a simpler special case. In our case, one should note that our large-scale simulation approach led to a structurally and numerically compact ensemble of similar solutions within the high-dimensional parameter space of the system. Given that we determined the ensemble with Monte Carlo simulations that cast a very wide net over the parameter space, it is difficult to imagine entirely different parameterizations that would capture all data as well as our ensemble and perform well in the validation studies we performed.

Moreover, considering that the available data were obtained from several independent transgenics, and that the stoichiometric system of the system is underdetermined, the likelihood of significantly other solutions appears to be rather small. Also, our simulations show that the system converges to the same steady-state starting from a wide array of initial conditions. Some arbitrary initial conditions actually lead to steady-state values outside of the defined physiological bounds; however, among the initial conditions that lead to admissible steady-states, several rounds of screening showed identical results.

In summary, it is well understood that model design is an iterative procedure, and while our logical analysis of numerical results suggested the step-wise addition or elimination of new features, there is no mathematical proof that the model ensemble is truly unique.

Outside these purely mathematical arguments, we might also look at the biological reasonableness of the model. For instance, one could ask why only CCR was subjected to substrate competition, while there are other shared enzymes. The answer is a matter of simplicity, as suggested by Ockham’s razor. Namely, we demonstrate that the substrate competition of CCR is needed to match the available data, while additional mechanisms are not necessary to explain the experimental data. Thus, we cannot exclude that additional regulatory mechanisms might exist, but we would need additional, independent data to confirm or refute such a hypothesis.

We also note that, although the model design progressed iteratively, we carefully investigated the necessity of including each individual mechanism a posteriori. For example, upon discovering that competitive inhibition over CCR improves H-lignin accumulation, we asked whether product inhibition was still vital for the model to explain the observations. We examined this hypothesis and determined that H-lignin accumulation could not be captured anymore. We therefore concluded that both mechanisms, product inhibition and CCR competition, are necessary. We found this conclusion reasonable, as both product inhibition and substrate competition are common in metabolic pathway systems.

### Model validation

The model with parameter values described above was constructed based on experimental data from wild-type switchgrass and four transgenic lines (4CL, CCR1, CAD and COMT knock-downs). To validate the model, experimental data from a separate transgenic plant, which had not been used in any way during the model design, were used to investigate how well the system performs under untested conditions. Namely, in a recent study, the transcription inhibitor PvMYB4 was over-expressed in order to reduce enzyme expression in the lignin pathway [19]. While metabolite concentrations were not measured for any of the pathway intermediates, the published data contain H, G and S lignin levels, as well as comparisons of enzyme activities between the wild type and PvMYB4 plants. The overall result of the study is a global reduction in the expression of the enzymes of the pathway, which in turn leads to 40–70 % decreases in total lignin.

**Fig. 6:**
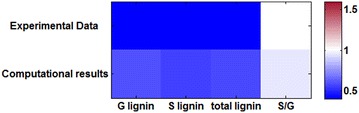
Fold changes in lignin monomer concentrations in PvMYB4 transgenic plants. The *top row* represents the average of PvMYB4 plants experimental data normalized with respect to the average of the control plants. The *second row* represent the results of the model with settings corresponding to the PvMYB4 experiment in [19], normalized with respect to wild-type model results. Wild type is set to 1, which corresponds to *white* in the *color bar*. H lignin only counts for 3 % of total lignin and is not shown in here

**Fig. 7:**
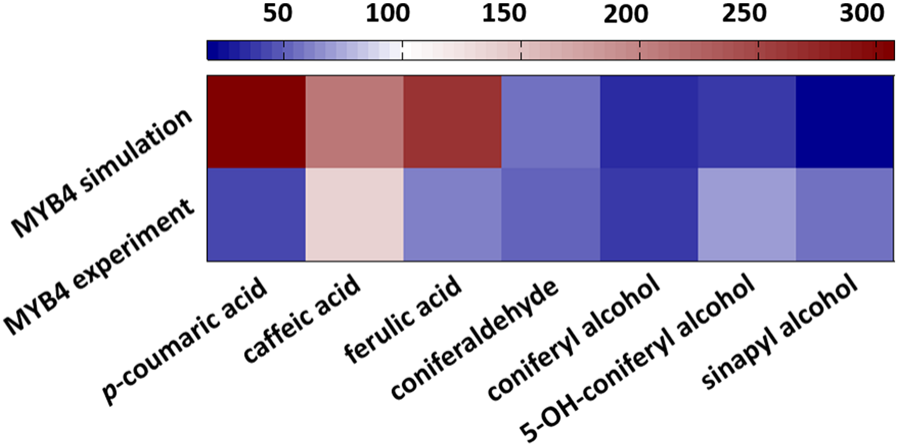
Steady-state profiles of key pathway metabolites in PvMYB4 overexpression as predicted by the model. Concentrations are normalized and the base value is set to 100, which corresponds to *white* in the *color bar*. Any increases with respect to the wild-type steady state are reflected in the *red* spectrum and any decreases in the *blue* spectrum

We tested our model against the profile of observed enzyme expression under overexpression of PvMYB4. We started with the already parameterized model without introducing any alterations or adjustments, except for resetting the appropriate enzyme activities, and tested how the system responded to the inhibition in comparison to the in vivo experiments [19]. Encouragingly, the altered G- and S-lignin amounts and their ratio, reported in the experimental study, are captured by the model with the compatible topological configuration quite well. The H-lignin was essentially unchanged in the experiment, while it slightly decreases in our model, in accordance with the data we used. However, H-lignin constitutes only about 3 % of the total lignin so that this difference is of no particular pertinence. Results are shown in Figs. 6 and 7. Figure 6 compares the fold change in lignin monomers between the experimental data and model results. The first row shows the fold change in G, S, the total lignin, and the S/G ratio comparing the wild type and PvMYB4 lines from the experiment; the second row corresponds to the computed configuration. As can be seen, the model results are quite consistent with experimental data.

This independent validation is very reassuring, especially with respect to future attempts to use metabolic engineering techniques to alter the S/G ratio in switchgrass. For instance, if further model predictions prove similarly reliable, the model could be used to simulate and optimize the outcome of combinatorial knockdowns, whose outcomes are not necessarily predictable with intuition alone. Such predictions would be very valuable, as a comprehensive combinatorial screening of double and triple knock-downs would neither be economical nor experimentally feasible.

While the published PvMYB4 data used for the first validation do not contain intermediate metabolite concentrations, a more recent study provides steady-state data for several of the pathway metabolites [20]. Comparing the published data in [20] with those in our model, we find that seven metabolites are represented in both, namely, caffeic acid, 5-OH-coniferyl alcohol, ferulic acid, sinapyl alcohol, coniferaldehyde, *p*-coumaric acid and coniferyl alcohol.

Figure 7 exhibits a comparison of the steady-state profiles. The top row shows the simulation results, while the bottom row represents experimentally measured steady-state concentrations in PvMYB4 normalized to wild type from [20]. The wild-type value for each concentration is set to 100 (white), and the red-blue spectrum represents increases or decreases in steady-state values of knockdowns. For five of these seven metabolites, our computational results of PvMYB4 conditions show the same semi-quantitative behavior in steady-state concentrations compared to the wild type; these are caffeic acid, 5-OH-coniferyl alcohol, sinapyl alcohol, coniferaldehyde and coniferyl alcohol. Discrepancies are seen in ferulic acid and *p*-coumaric acid. Here, the experimental data show a decrease in the steady-state concentrations, while our computational results predict an accumulation. Interestingly, these differences occur for metabolites whose effluxes out of the lignin pathway are ill defined, because their characteristics were not documented in the literature. It is therefore likely that they are not optimally parameterized in the model.

## Discussion

In this work, we developed an ensemble of models of lignin biosynthesis in stem and tiller tissue in switchgrass, *P. virgatum*. The model reflects the consequences of various enzyme knock-downs quite well and performed satisfactorily in two validation studies with experimental data that had not been used in the model design or implementation. We used as the modeling framework the generalized mass action (GMA) format within biochemical systems theory (BST) [21–25]. The power-Law representation, which is the hallmark of this type of model, is arguably the least biased default formulation and by its mathematical nature avoids problems due to possibly invalid assumptions that may cast doubt on traditional Michaelis–Menten models in vivo [26]. Parameter values were, as always, difficult to obtain in a direct manner. We used for this purpose experimental knock-down data and a sophisticated Monte Carlo sampling strategy that has been used very successfully for similar systems before [14]. As a particular sub-goal, we investigated the regulatory mechanism of the pathway and the possible co-localization or coupling of the pair of enzymes, CCR1/CAD that was previously suggested for *Medicago* [5].

To elucidate the co-localization or coupling of these enzymes in switchgrass, we studied multiple configurations that seemed a priori plausible and identified those natural designs that were consistent with the experimental data. The consistent designs were further examined under different regulation scenarios. The main result from this study is a very robust model of lignin biosynthesis in switchgrass that is consistent with all available data. The model was, at least to some degree, validated with a formerly unused dataset. If this validation can be confirmed and expanded experimentally, the model proposed here may be used to predict responses of the natural pathway system to alterations that are difficult to assess with experimental means. For instance, a further validated model will allow the prediction of responses to combinatorial knockdowns that could be the basis for future designs of more sophisticated transgenic lines than are currently available.

The computational analysis suggests the co-localization or functional coupling of the two enzymes CCR1 and CAD. Metabolic channeling and compartmentalization in plants have been identified in many biochemical pathways [27]. Of importance here, it has been suggested that enzymes catalyzing early reactions in the monolignol pathway may be co-localized in their binding to the ER. For instance, a multi-protein complex has been identified between PAL and C4H, and it seems that most of the substrates use these channels, but that some substrate undergoes the metabolic conversion in two steps [28–30]. C4H can also form a complex with C3′H [31], and it has been suggested that different forms of 4CL form a complex in poplar [32]. Independent computational work on alfalfa came to a similar conclusion for channeling of enzymes associated with coniferaldehyde, which were proposed to form a metabolic channel [14]. Our results on switchgrass, presented in this article, are in line with the latter result and suggest moreover that channeling around coniferaldehyde is necessary to capture the available data.

**Fig. 8:**
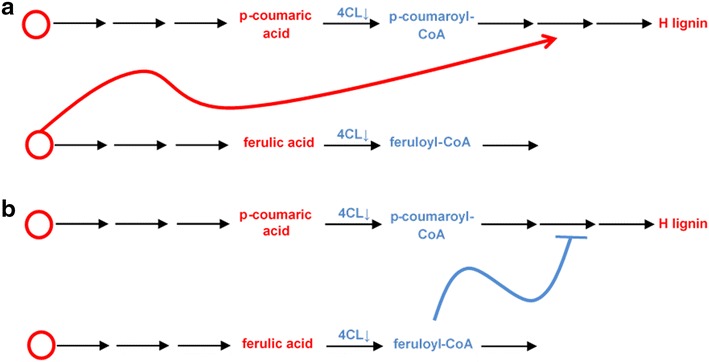
Two plausible explanations for an increase in the H lignin concentration in 4CL transgenic lines. **a** represents a putative increase in an activator located *upstream* of the enzyme 4CL, whereas **b** shows a putative decrease in an inhibitor located *downstream* of 4CL
- There exists an inhibitor for the H lignin branch. This metabolite would have to be located such that its concentration is decreased due to the 4CL knockdown, which means that the inhibitor activity is inhibited and therefore exerts a net positive effect on the system (Fig. 8b). Feruloyl-CoA could be a good candidate for this scenario.

The comparative study of different configurations revealed that consistency with the available experimental data was most difficult to achieve for transgenic 4CL down-regulated lines, in which, surprisingly, the H lignin concentration is increased. This observation is at first counterintuitive because 4CL is located directly upstream of the H lignin precursors, which would lead to the a priori expectation of a decrease in H lignin. The combination of two postulated types of regulatory mechanisms was able to explain this observation. The first is product inhibition, which is observed quite frequently in biochemical systems. While improving the data compatibility, this mechanism turned out to be insufficient, thus requiring additional signaling. Arguably the simplest explanation is a regulatory structure that works in either of the mechanisms below:

- An intermediate in the pathway is increased in response to the 4CL knockdown and activates the precursors of H lignin synthesis. The most likely candidates for this scenario appear to be *p*-coumaric acid, caffeic acid, and ferulic acid (Fig. 8a).

The current literature does not support the first hypothesis. By contrast, multiple candidates are available for the second scenario. A reasonable scenario arises from the fact that the lignin pathway in switchgrass includes parallel fluxes that share the same enzymes. Indeed, 4CL, CAD, COMT, F5H and CCR1 all catalyze multiple reactions, and it is likely that the substrates exert competitive inhibition for the shared enzyme, as it was also suggested in [33]. Supporting this scenario, a targeted numerical analysis demonstrated that competition over CCR1 perfectly matches the results of the 4CL knockdown line in the model with product inhibition. One could surmise that the latter mechanism would suffice to represent the increase in H lignin concentration. To test this hypothesis, we simulated the model with enzyme competition but without product inhibition. The results showed that competitive inhibition by itself could not satisfactorily resolve the issue. By contrast, the combined model containing product inhibition and competitive inhibition matches the experimental results very well. One should also recall that the product inhibition and substrate competition mechanisms only work properly if the proposed metabolic channel is present (Fig. 3, Configuration 1).

**Fig. 9:**
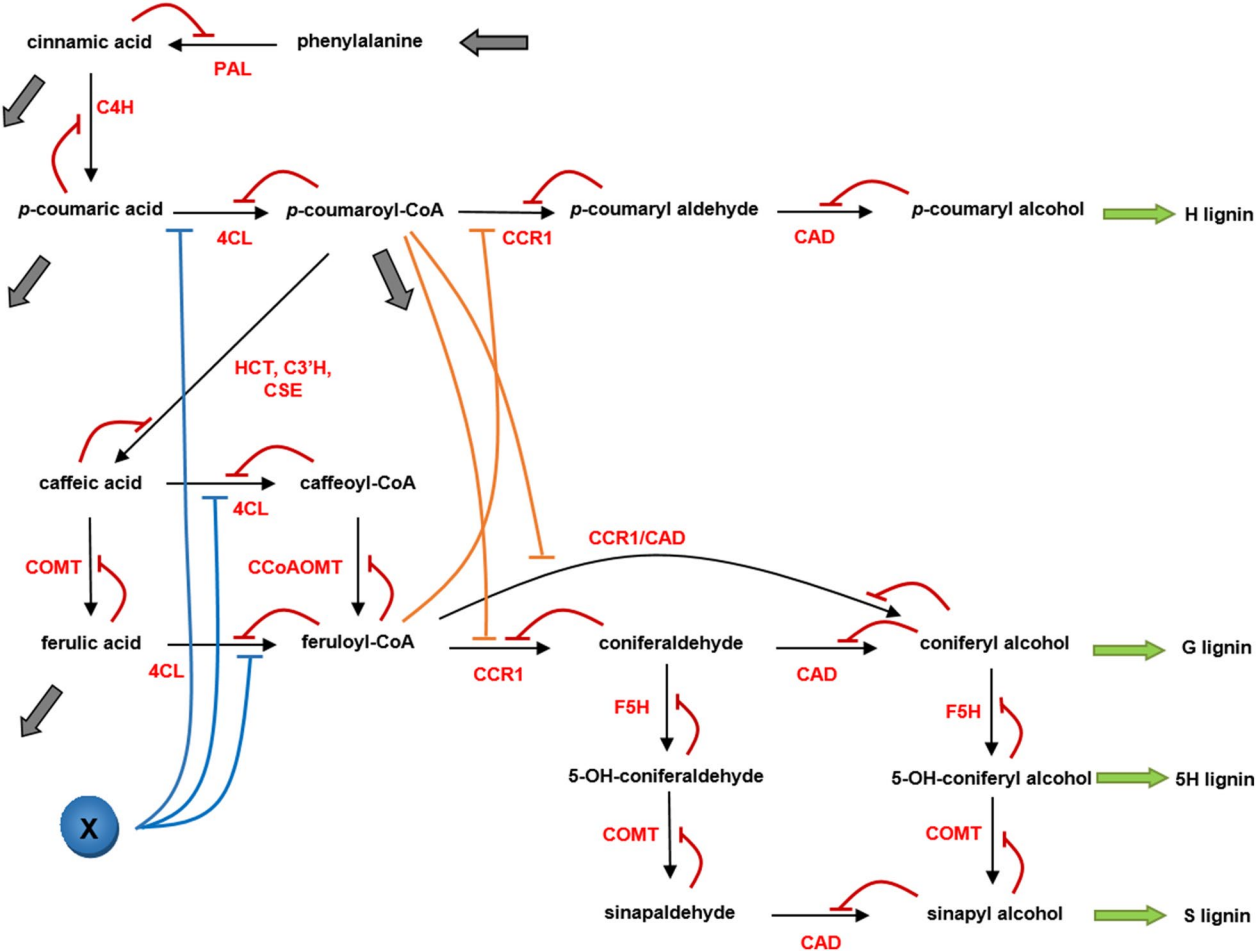
Full scheme of the lignin biosynthetic pathway in switchgrass suggested by the computational results of this study. All regulatory signals, i.e., universal product inhibition, substrate competition over CCR1, and 4CL inhibition are shown. The 4CL inhibiting agent is unknown and therefore denoted with X. 5-OH-ferulic acid might be a candidate for this role

Another aspect of the experimental data that was not captured well by the original model, even when product inhibition and substrate competition over CCR1 were taken into account, is the accumulation of 4CL substrates in COMT transgenic plants. Particularly counterintuitive appears to be the accumulation of ferulic acid as a product of a reaction catalyzed by COMT. The observed concomitant decrease in the steady-state concentration of coniferaldehyde supports the possible explanation that the observation is due to regulation that begins to inhibit the conversion of ferulic acid into coniferaldehyde, when 4CL substrates are in excess. The simultaneous accumulation of *p*-coumaric acid and caffeic acid provides additional evidence that reactions catalyzed by 4CL are inhibited in COMT knockdown plants. Accounting for this feature to our model, all experimental data are represented well. The mechanism of the regulation remains a subject of further experimental investigations. Figure 9 shows the pathway including all inferred regulatory signals.

## Conclusions

The model proposed in this article captures all available data and performed well in independent PvMYB4 validation experiments. This good match with data is reason for cautious optimism, which however is to be supported with further experimental confirmation. Indeed, work is in progress to generate and analyze additional transgenic switchgrass lines and to incorporate further lignin compositional and enzyme activity and kinetic data into the model. If the model fares well in these additional validation studies, the results from the present study suggest that one might use the model for predictions, for instance, with respect to double knock-downs, and for optimization studies that could potentially affect the lignin-based recalcitrance in switchgrass in a favorable manner.

## Methods

### Model construction

Much of the analysis in this article consists of comparisons and simulations with different models. Each of these models consists of a system of differential equations that represent the rate of change in metabolite concentrations, which are represented as dependent variables. The right-hand side of each equation contains a set of fluxes which enter (influxes) or leave (effluxes) the metabolite pool. Enzymes are included in the model as independent variables; that is, they do not change in activity during any given computational experiment. The generic formulation of each equation is

2 $$\frac{{{\rm d}X_{i} }}{{\rm d}t} = \sum\limits_{j = 1}^{k} {s_{i,j} V_{j} }$$

where each *X*_*i*_ is a metabolite, *V*_*j*_ are fluxes associated with *X*_*i*_, and the quantities *s*_*i,j*_ are stoichiometric coefficients, which here are simply 0, 1 or −1 and determine whether flux *V*_*j*_ affects *X*_*i*_ as influx or efflux or not at all. Each *V*_*j*_ is a function of some or potentially all of the *X*_*i*_. At the steady state, the left-hand side is equal to zero, and fluxes can be assessed with methods of linear algebra [34]. Because the system in our case is underdetermined, infinitely many solutions satisfy the steady-state condition. Following the tenets of Flux Balance Analysis (FBA), an objective function is chosen and the problem is solved as a linear programming problem [34]. In the present study, maximizing the total amount of lignin is set as the objective of the system. The optimization problem is solved using MATLAB (version R2014a, The MathWorks, Natick, MA, USA) function *linpro*. The output is the set of fluxes at the steady state that maximizes the defined objective.

The fluxes themselves are formulated as general mass action (GMA) models of the type

3 $$V_{j} = \alpha_{j} \prod\limits_{r = 1}^{n} {X_{r}^{{g_{r,j} }} } \prod\limits_{r = n + 1}^{n + m} {X_{r}^{{h_{r,j} }} }$$

within the modeling framework of BST [21, 22, 24, 35, 36]. Here, *α*_*j*_ is the rate constant, each *X*_*r*_, for 1 < *r* < *n*, is a metabolite or, for *n* + 1 < *r* < *n* + *m*, an enzyme involved in the reaction. Thus, *n* is the number of metabolites and *m* is the number of enzymes in the pathway. The exponents *g*_*r,j*_ are kinetic orders that quantify the effect of *X*_*r*_ on *V*_*j*_. Similarly, *h*_*r,j*_ describes the effect of the enzyme on the reaction. It is customary to set each *h*_*r,j*_ to 0 or 1, thus merely reflecting absence or presence of an enzyme in a specific flux. This setting of *h*_*r,j*_ = 1 is consistent with the underpinnings of Michaelis–Menten, mass-action, and other traditional models, where a reaction is assumed to be a linear function of enzyme activity. All other kinetic orders *g*_*r,j*_ are sampled from the range between 0 and 1 if *X*_*r*_ is a substrate or activator of the flux, or from the range between −1 and 0 if *X*_*r*_ is an inhibitor.

Due to the nature of the present experimental data for switchgrass, the real concentrations of metabolites and enzyme activities in vivo are unknown. As a remedy, we normalize these quantities with respect to the steady state and set all base values to 100. Thus, we set

4 $$Z_{i} = \frac{{100X_{i} }}{{X_{SS,i} }}$$

and express Eq. (4) as

5 $$\frac{{{\rm d}Z_{i} }}{{\rm d}t} = \frac{100}{{X_{SS,i} }}\frac{{{\rm d}X_{i} }}{{\rm d}t} = \frac{100}{{X_{SS,i} }}\sum\limits_{j = 1}^{k} {s_{i,j} V_{j} }$$

Since the constant *X*_*ss,i*_ refers to the steady state, simple algebra adjusts the rate constants to this steady state. Thus, we obtain

6 $$\frac{{{\rm d}Z_{i} }}{{\rm d}t} = \sum\limits_{j = 1}^{k} {s_{i,j} \alpha_{j} \prod\limits_{r = 1}^{n} {\left( {\frac{{100X_{r} }}{{X_{SS,r} }}} \right)^{{g_{r,j} }} } } \prod\limits_{r = n + 1}^{n + m} {\left( {\frac{{X_{r} }}{{X_{SS,r} }}} \right)^{{h_{r,j} }} }$$

The enzymes are independent variables and therefore constant for each experiment. Therefore, *X*_*r*_ = *X*_*ss*_ for *n* + 1 < *r* < *n* + *m* for wild type, whereas for a transgenic line it takes a value between 0 and 1, according to the level of knockdown. At the steady state we have:

7 $$\begin{aligned} 0 &= \sum\limits_{j = 1}^{k} {s_{i,j} \alpha_{j} \prod\limits_{r = 1}^{n} {\left( {\frac{{100X_{r} }}{{X_{SS,r} }}} \right)^{{g_{r,j} }} } } \hfill \\ 0 &= \sum\limits_{j = 1}^{k} {s_{i,j} \alpha_{j} \prod\limits_{r = 1}^{n} {100^{{g_{r,j} }} } } \hfill \\ \end{aligned}$$

with this setting, each steady-state flux is given as

8 $$V_{j} = \alpha_{j} \prod\limits_{r = 1}^{n} {100^{{g_{r,j} }} } = \alpha_{j} 100^{{\sum\limits_{r = 1}^{n} {g_{r,j} } }} .$$

If the flux is known, the rate constant can be computed as

9 $$\alpha_{j} = V_{j} /100^{{\sum\limits_{r = 1}^{n} {g_{r,j} } }} .$$

With these settings, the set of the differential equations for the model takes the form below.

10 $$\begin{aligned} &\frac{{{\rm d}Z_{1} }}{{\rm d}t} = I_{1} - V_{1} \quad\quad\quad\quad\quad\quad\quad\;\; \frac{{{\rm d}Z_{9} }}{{\rm d}t} = V_{12} - V_{14} - V_{18}\\ &\frac{{{\rm d}Z_{2} }}{{\rm d}t} = V_{1} - V_{2} - V_{3} \quad\quad\quad\quad\;\;\; \frac{{{\rm d}Z_{10} }}{{\rm d}t} = V_{13} + V_{14} - V_{15} - V_{26}\\ &\frac{{{\rm d}Z_{3} }}{{\rm d}t} = I_{2} + V_{3} - V_{4} - V_{8} \quad\quad\quad \frac{{{\rm d}Z_{11} }}{{\rm d}t} = V_{15} - V_{16} - V_{19}\\ &\frac{{{\rm d}Z_{4} }}{{\rm d}t} = V_{4} - V_{5} - V_{9} - V_{10} \quad\quad\; \frac{{{\rm d}Z_{12} }}{{\rm d}t} = V_{16} + V_{26} - V_{17} - V_{20} \\ &\frac{{{\rm d}Z_{5} }}{{\rm d}t} = V_{5} - V_{6} \quad \quad\quad\quad\quad\quad\;\;\; \frac{{{\rm d}Z_{13} }}{{\rm d}t} = V_{19} - V_{22}\\ &\frac{{{\rm d}Z_{6} }}{{\rm d}t} = V_{6} - V_{7} \quad\quad\quad\quad\quad\quad\;\;\; \frac{{{\rm d}Z_{14} }}{{\rm d}t} = V_{20} - V_{21} - V_{23}\\ &\frac{{{\rm d}Z_{7} }}{{\rm d}t} = V_{9} - V_{11} - V_{12} \quad\quad\quad\quad \frac{{{\rm d}Z_{15} }}{{\rm d}t} = V_{22} - V_{24}\\ &\frac{{{\rm d}Z_{8} }}{{\rm d}t} = V_{11} - V_{13} \quad\quad\quad\quad\quad\quad \frac{{{\rm d}Z_{16} }}{{\rm d}t} = V_{23} + V_{24} - V_{25}\\ \end{aligned}$$

where the quantities $I_{i}$ include the influxes into the pathway and the fluxes, $V_{i}$, are defined as follows:

11 $$\begin{aligned} &V_{1} = \alpha_{1} Z_{1}^{{g_{1,1} }} Z_{2}^{{g_{2,1} }} Z_{17}^{{}} \quad\quad\quad\quad\; V_{14} = \alpha_{14} Z_{9}^{{g_{9,14} }} Z_{10}^{{g_{10,14} }} Z_{20}^{{}}\\ &V_{2} = \alpha_{2} Z_{2}^{{g_{2,2} }} Z_{18} \quad\quad\quad\quad\quad\quad V_{15} = \alpha_{15} Z_{10}^{{g_{10,15} }} Z_{11}^{{g_{11,15} }} Z_{4}^{{g_{4,15} }} Z_{21}^{{}}\\ &V_{3} = \alpha_{3} Z_{2}^{{g_{2,3} }} Z_{3}^{{g_{3,3} }} Z_{19}^{{}} \quad\quad\quad\quad\; V_{16} = \alpha_{16} Z_{11}^{{g_{11,16} }} Z_{12}^{{g_{12,16} }} Z_{22}^{{}}\\ &V_{4} = \alpha_{4} Z_{3}^{{g_{3,4} }} Z_{4}^{{g_{4,4} }} Z_{20}^{{}} \quad\quad\quad\quad\; V_{17} = \alpha_{17} Z_{12}^{{g_{12,17} }} Z_{29}^{{}}\\ &V_{5} = \alpha_{5} Z_{4}^{{g_{4,5} }} Z_{5}^{{g_{5,5} }} Z_{10}^{{g_{10,5} }} Z_{21}^{{}} \quad\quad\; V_{18} = \alpha_{18} Z_{9}^{{g_{9,18} }} Z_{30}^{{}}\\ &V_{6} = \alpha_{6} Z_{5}^{{g_{5,6} }} Z_{6}^{{g_{6,6} }} Z_{22}^{{}} \quad\quad\quad\quad\; V_{19} = \alpha_{19} Z_{11}^{{g_{11,19} }} Z_{13}^{{g_{13,19} }} Z_{31}^{{}}\\ &V_{7} = \alpha_{7} Z_{6}^{{g_{6,7} }} Z_{23}^{{}} \quad \quad\quad\quad\quad\quad V_{20} = \alpha_{20} Z_{12}^{{g_{12,20} }} Z_{14}^{{g_{14,20} }} Z_{31}^{{}}\\ &V_{8} = \alpha_{8} Z_{3}^{{g_{3,8} }} Z_{24}^{{}} \quad\quad\quad\quad\quad\quad V_{21} = \alpha_{21} Z_{14}^{{g_{14,21} }} Z_{32}^{{}}\\ &V_{9} = \alpha_{9} Z_{4}^{{g_{4,9} }} Z_{7}^{{g_{7,9} }} Z_{25}^{{}} \quad\quad\quad\quad\; V_{22} = \alpha_{22} Z_{13}^{{g_{13,22} }} Z_{15}^{{g_{15,22} }} Z_{27}^{{}}\\ &V_{10} = \alpha_{10} Z_{4}^{{g_{4,10} }} Z_{26}^{{}} \quad\quad\quad\quad\quad V_{23} = \alpha_{23} Z_{14}^{{g_{14,23} }} Z_{16}^{16,23} Z_{27}^{{}} \\ &V_{11} = \alpha_{11} Z_{7}^{{g_{7,11} }} Z_{8}^{{g_{8,11} }} Z_{20}^{{}} \quad\quad\quad V_{24} = \alpha_{24} Z_{15}^{{g_{15,24} }} Z_{16}^{{g_{16,24} }} Z_{22}^{{}}\\ &V_{12} = \alpha_{12} Z_{7}^{{g_{7,12} }} Z_{9}^{{g_{9,12} }} Z_{27}^{{}} \quad\quad\quad V_{25} = \alpha_{25} Z_{16}^{{g_{16,25} }} Z_{33}^{{}}\\ &V_{13} = \alpha_{13} Z_{8}^{{g_{8,13} }} Z_{10}^{{g_{10,13} }} Z_{28}^{{}}\quad\quad\;\;\; V_{26} = \alpha_{26} Z_{10}^{{g_{10,26} }} Z_{12}^{{g_{12,26} }} Z_{4}^{{g_{4,26} }} Z_{34}^{{}}\\ \end{aligned}$$

The metabolites of the pathway are

12 $$\begin{aligned} Z_{1} &:{\text{phenylalanine}} \quad\quad\quad\quad\;\;\; Z_{9} :{\text{ferulic acid}}\\ Z_{2} &:{\text{cinnamic acid}} \quad\quad\quad\quad\;\; Z_{10} :{\text{feruloyl-CoA}}\\ Z_{3} &:{\text{p-coumaric acid}}\quad\quad\quad\;\;\; Z_{11} :{\text{coniferaldehyde}}\\ Z_{4} &:p{\text{-coumaroyl CoA}} \quad\quad\quad Z_{12} :{\text{coniferyl alcohol }}\\ Z_{5} &:p{\text{-coumaryl aldehyde }} \quad\;\; Z_{13} : 5 {\text{-OH-coniferaldehyde}}\\ Z_{6} &:p{\text{-coumaryl alcohol}} \quad\quad\;\; Z_{14} : 5 {\text{-OH-coniferyl alcohol}}\\ Z_{7} &:{\text{caffeic acid}} \quad\quad\quad\quad\quad\;\;\; Z_{15} :{\text{sinapaldehyde}}\\ Z_{8} &:{\text{caffeoyl CoA}} \quad\quad\quad\quad\quad Z_{16} :{\text{sinapyl alcohol}}\\ \end{aligned}$$

while the enzymes of the pathway are

13 $$\begin{aligned} Z_{17}^{{}} &:{\text{PAL, }}\,{\text{L-phenylalanine ammonia-lyase}} \\ Z_{19}^{{}} &:{\text{C4H, }}\,{\text{cinnamate 4-hydroxylase}} \\ Z_{20}^{{}} &: 4 {\text{CL, }}\, 4 {\text{-coumarate:CoA ligase}} \\ Z_{21}^{{}} &:{\text{CCR1, }}\,{\text{cinnamoyl CoA reductase}} \\ Z_{22}^{{}} &:{\text{CAD, }}\,{\text{cinnamyl alcohol dehydrogenase}} \\ {\text{Z}}_{ 2 5} &:{\text{HCT,}}\,\,{\text{hydroxycinnamoyl-CoA:shikimate hydroxycinnamoyl transferase/}} \\ &\quad {\text{C3}}'{\text {H}},\,\,p{\text{-coumaroyl shikimate 3}}'{\text{-hydroxylase/}} \\ &\quad{\text{CSE,}}\,\,{\text{c}}\,{\text{affeoyl shikimate esterase}} \\ Z_{27}^{{}} &:{\text{COMT, }}\,{\text{caffeic acid}}\, O{\text{-methyltransferase}} \\ {\text{Z}}_{ 2 8} &:{\text{CCoAOMT, }}\,{\text{caffeoyl CoA}} \, O{\text{-methyltransferase}} \\ Z_{31}^{{}} &:{\text{F5H, ferulate 5-hydroxylase}} \end{aligned}$$

**Fig. 10:**
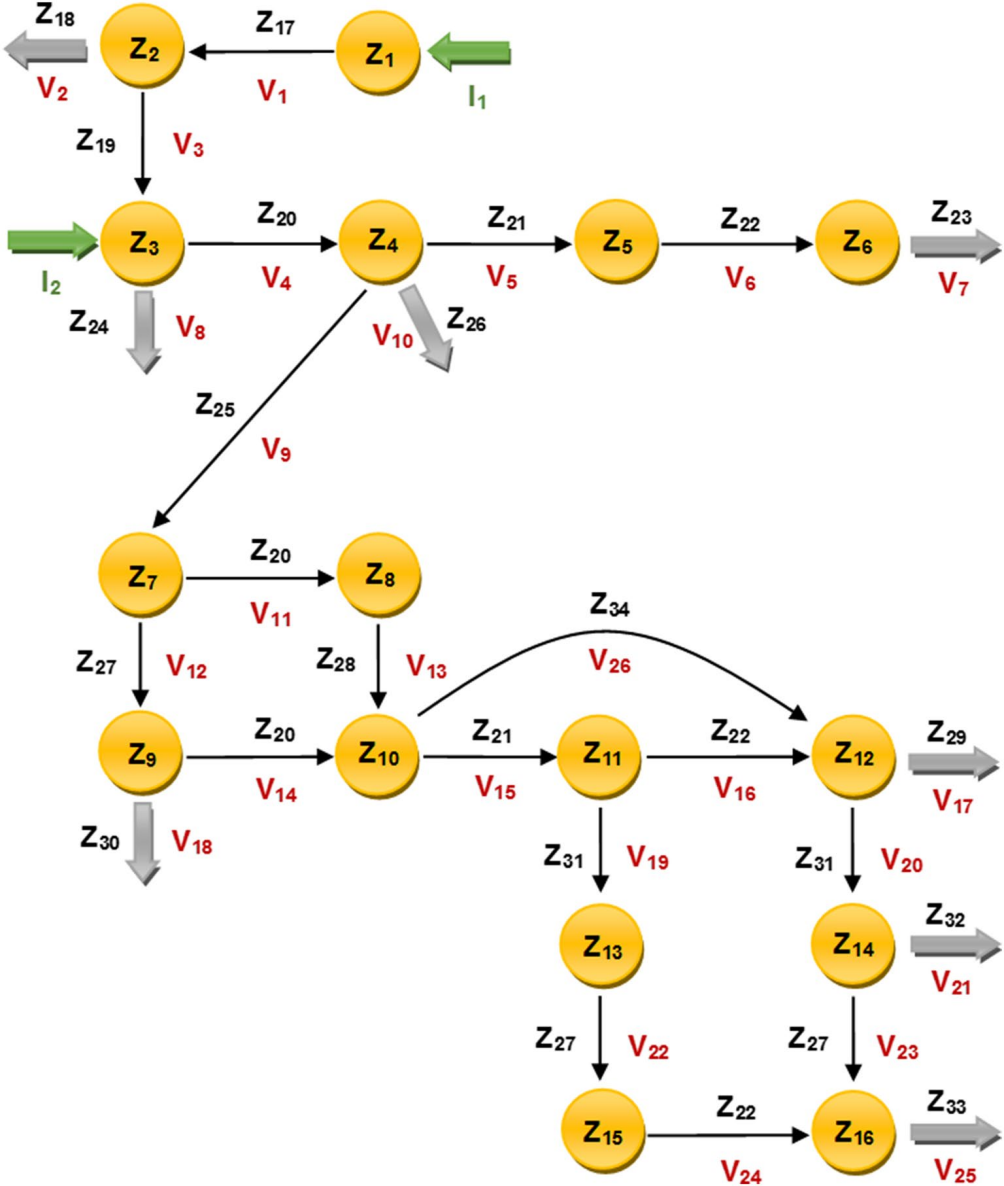
Lignin pathway in the notation of the model. Redundancy of enzymes, i.e., 4CL, CCR1, CAD, COMT and F5H in parallel fluxes reduces the dimension of state space. The enzymes HCT, C3′H and CSE in flux V_9_ are merged into one independent variable, Z_25_. Note that the presence of the G-channel, V_26_, is an inference from the computational simulations results

Note that the model does not account for the dynamics of tyrosine, which we consider constant here. The model scheme is shown in Fig. 10.

### Parameter space and sampling

Similar to earlier work [14, 37, 38], flux rates are computed with FBA. Next, the parameters to be estimated are the kinetic orders and rate constants are in turn estimated from the FBA results and randomly sampled kinetic orders through the steps mentioned above. The kinetic order of a metabolite is positive if the metabolite is a substrate or activator of the flux and negative if it acts as an inhibitor. The kinetic order of each enzyme has a default value of 1, which is in line with traditional enzyme kinetics, because it is customary to assume that a flux has a linear relationship with the enzyme. This assumption is explicitly or implicitly made in essentially all traditional models of enzyme kinetics as, for instance, in the Michaelis–Menten formalism, where *V*_max_ equals *k*_cat_ times the enzyme concentration.

The down-regulation of an enzyme is modeled through the enzyme concentration, not the kinetic order. Since the concentrations of metabolites and enzymes are normalized, the concentration of an enzyme in the wild type has the default value of 1. In transgenics, the concentration of the corresponding enzyme is set to a value less than one if it is down-regulated. For example, to represent the 4CL knockdown, the concentration of the enzyme is set to 0.6 as the enzyme is down-regulated by 40 %.

To account for product inhibition, the inhibiting product is represented in each reaction by a factor consisting of its concentration, raised to a negative power. The result is as follows:

14 $$V = \alpha S^{{g_{S} }} P^{{g_{I} }} ,\,\,\,\,\,\,\, - 1 < \frac{{g_{I} }}{{g_{S} }} < 0$$

Here, *S* is the substrate, *P* is the product, *g*_*I*_ is the kinetic order of the inhibiting product and *g*_*S*_ is the kinetic order of the substrate. The ratio of kinetic orders could be derived directly [22] from the corresponding expression for a product-inhibited Michaelis–Menten reaction, which takes the form

15 $$V = \frac{{V_{\hbox{max} } \frac{S}{{K_{m} }}}}{{1 + \frac{S}{{K_{m} }} + \frac{P}{{K_{I} }}}}$$

The power-law form of Eq. 15 can directly be computed from the tenets of Biological Systems Theory (BST), which defines the kinetic orders as

16 $$\begin{aligned}g_{S} = \left. {\frac{\partial V}{\partial S} \cdot \frac{S}{V}} \right|_{\rm {OP}} = \left. {\frac{{1 + \frac{P}{{K_{I} }}}}{{1 + \frac{S}{{K_{m} }} + \frac{P}{{K_{I} }}}}} \right|_{\rm {OP}}\\ g_{I} = \left. {\frac{\partial V}{\partial P} \cdot \frac{P}{V}} \right|_{\rm {OP}} = \left. {\frac{{ - \frac{P}{{K_{I} }}}}{{1 + \frac{S}{{K_{m} }} + \frac{P}{{K_{I} }}}}} \right|_{\rm {OP}} \,\, \end{aligned}$$

Rearrangement of these equations gives the ratio of kinetic orders as follows:

17 $$- 1 < \left. {\frac{{g_{I} }}{{g_{S} }} = \frac{ - P}{{P + K_{I} }}} \right|_{\rm {OP}} < 0$$

The bounded ratio of kinetic orders provides a valuable constraint for the Monte Carlo simulations, because a fixed ratio does not affect the dimension of the parameter space.

For the initial set of simulations, the sampling space is chosen as a unit hypercube in ℝ$^{n}$ where *n* is number of kinetic orders to be estimated. A set of 100,000 parameter sets is generated for each scenario simulation. 10,000 sets are randomly generated from the sampling space using Latin Hypercube Sampling to assure a homogeneous coverage of the space, while 90,000 sets are generated by the MATLAB (version R2014a, The MathWorks, Natick, MA, USA) function *rand*. Each parameter set is simulated to examine whether the model with this set can match the experimental results for the wild type and transgenics. The model is deemed a match for the experimental results if:

- The model returns proper lignin contents and S/G ratios for the wild type and different transgenics, with down-regulation of 4CL (40 %), CCR1 (50 %), COMT (30 %), and CAD (30 %).
- The model returns the proper decrease in lignin content in the case of knockdowns in 4CL, CCR1, COMT, and CAD.
- The model demonstrates an increase in H lignin in 4CL transgenics.
- The model matches the altered metabolite concentrations in the COMT transgenic.

If a parameter satisfies the above conditions, it is recorded along with the corresponding topological configuration.

While our model approach emphasizes ensembles of feasible models, the parameter values in Tables 2, 3, and 4 represent one implementation, which we used for further numerical exploration. This specific parameter set corresponds to the minimum error in the comparison of the model results in PvMYB4 and the experimental data.

**Table 2:** A sample of rate constants from the ensemble of rate constants

$\alpha_{1}$	0.5233	$\alpha_{8}$	0.0058	$\alpha_{15}$	0.0771	$\alpha_{22}$	0.0392
$\alpha_{2}$	0.1053	$\alpha_{9}$	0.2265	$\alpha_{16}$	0.0881	$\alpha_{23}$	0.1573
$\alpha_{3}$	0.15	$\alpha_{10}$	0.0024	$\alpha_{17}$	0.0168	$\alpha_{24}$	0.0712
$\alpha_{4}$	0.2711	$\alpha_{11}$	0.1054	$\alpha_{18}$	0.002	$\alpha_{25}$	0.1154
$\alpha_{5}$	0.1832	$\alpha_{12}$	0.1095	$\alpha_{19}$	0.2212	$\alpha_{26}$	0.0814
$\alpha_{6}$	0.003	$\alpha_{13}$	0.1452	$\alpha_{20}$	0.0402		
$\alpha_{7}$	0.0042	$\alpha_{14}$	0.2681	$\alpha_{21}$	0.0002

**Table 3:** A sample of kinetic orders from the ensemble of kinetic orders

$g_{1,1}$	0.2813	$g_{6,7}$	0.4040	$g_{10,14}$	−0.1023	$g_{14,21}$	0.8535
$g_{2,1}$	−0.1406	$g_{3,8}$	0.5759	$g_{10,15}$	0.9009	$g_{13,22}$	0.7673
$g_{2,2}$	0.0846	$g_{4,9}$	0.6118	$g_{11,15}$	−0.4505	$g_{15,22}$	−0.3836
$g_{2,3}$	0.8240	$g_{7,9}$	−0.3059	$g_{4,15}$	−0.0355	$g_{14,23}$	0.1043
$g_{3,3}$	−0.4120	$g_{4,10}$	0.6398	$g_{11,16}$	0.5198	$g_{16,23}$	−0.0521
$g_{3,4}$	0.5669	$g_{7,11}$	0.6277	$g_{12,16}$	−0.2599	$g_{15,24}$	0.5080
$g_{4,4}$	−0.2835	$g_{8,11}$	−0.3138	$g_{12,17}$	0.7121	$g_{16,24}$	−0.2540
$g_{4,5}$	0.0710	$g_{7,12}$	0.6414	$g_{9,18}$	0.6982	$g_{16,25}$	0.2855
$g_{5,5}$	−0.0355	$g_{9,12}$	−0.3207	$g_{11,19}$	0.0160	$g_{10,26}$	0.7116
$g_{10,5}$	−0.4505	$g_{8,13}$	0.4885	$g_{13,19}$	−0.0080	$g_{12,26}$	−0.3558
$g_{5,6}$	0.9630	$g_{10,13}$	−0.2442	$g_{12,20}$	0.6973	$g_{4,26}$	−0.0355
$g_{6,6}$	−0.4815	$g_{9,14}$	0.2046	$g_{14,20}$	−0.3487

**Table 4:** Initial values

$Z_{0,1}$	100	$Z_{0,5}$	100	$Z_{0,9}$	100	$Z_{0,13}$	100
$Z_{0,2}$	100	$Z_{0,6}$	100	$Z_{0,10}$	100	$Z_{0,14}$	100
$Z_{0,3}$	100	$Z_{0,7}$	100	$Z_{0,11}$	100	$Z_{0,15}$	100
$Z_{0,4}$	100	$Z_{0,8}$	100	$Z_{0,12}$	100	$Z_{0,16}$	100

## Additional files

**Additional file 1.** Analysis of the lignin pathway with inclusion of caffeyl aldehyde.
**Additional file 2.** Principal component analysis.

## Abbreviations

PAL: l-phenylalanine ammonia-lyase
C4H: cinnamate 4-hydroxylase
4CL: 4-coumarate:CoA-ligase
CCR1: cinnamoyl CoA reductase
CAD: cinnamyl alcohol dehydrogenase
HCT: hydroxycinnamoyl-CoA:shikimate hydroxycinnamoyl transferase
C3′H: *p*-coumaroyl shikimate 3′-hydroxylase
CSE: caffeoyl shikimate esterase
COMT: caffeic acid *O*-methyltransferase
CCoAOMT: caffeoyl CoA *O*-methyltransferase
F5H: ferulate 5-hydroxylase
ER: endoplasmic reticulum
BST: biochemical systems theory
GMA: generalized mass action
PCA: principal component analysis
FBA: flux balance analysis
