# Supplementary material for: Computational inference of the structure and regulation of the lignin pathway in Panicum virgatum
Source: Biotechnol Biofuels. 2015 Sep 17;8:151. doi: 10.1186/s13068-015-0334-8 (PMC4574612; doi:10.1186/s13068-015-0334-8)
Supplement: Supplementary file 1 — Additional file 1. Analysis of the lignin pathway with inclusion of caffeyl aldehyde. [file 13068_2015_334_MOESM1_ESM.docx]

**Additional file 1**

**Computational Inference of the Structure and Regulation of the**

**Lignin Pathway in *Panicum virgatum***

Mojdeh Faraji, Luis L. Fonseca, Luis Escamilla-Treviño, Richard A. Dixon, Eberhard O. Voit

**Text S1. Analysis of the Lignin Pathway with Inclusion of Caffeyl Aldehyde.**

The lignin pathway in switchgrass is still not entirely understood. In particular, some species synthesize caffeyl aldehyde, but this does not seem to be the case in switchgrass. The account for this intermediate metabolite complicates the structural analysis, which is discussed in the following, because many more topological pathway configurations are possible.

Recent enzyme kinetic experiments suggest that a reaction catalyzed by CCR1 with caffeoyl CoA as substrate is not likely to occur in switchgrass. The *K_cat_*/*K_M_* values are up to 10 times higher for feruloyl CoA compared to caffeoyl CoA [[1](#_ENREF_1)]. However, because the *in vivo* concentration of caffeoyl CoA is unknown, the reaction could be plausible. The reason is that a higher steady-state concentration of caffeoyl CoA would compensate for the lower *K_cat_*/*K_M_* ratio, and a new metabolite, caffeyl aldehyde, would have to become a component of the pathway.

The most significant change in comparison with the results of the main text is that the COMT/F5H complex could constitute a second functional channel. As a consequence, the number of candidates for topological configurations jumps to 19, and large-scale simulations confirm that 12 among these are compatible with experimental data. Between the 12, four configuration are significantly more abundant in parameter space. Interestingly, all four include at least one channel. Results for the pathway containing caffeyl aldehyde are shown in Figures S1- S5. They correspond to the main result figures in the text. In addition, all theoretically admissible pathway configurations that include caffeyl aldehyde can be mapped according to their total numbers of reaction steps (Fig. S6). It is interesting to note that in this type of representation the compatible configurations are closely clustered, considering that the order of configurations in the second and third row is more or less arbitrary.

**Figure S1. Topological Configurations.** A set of 19 structures is plausible when CCR1/CAD and COMT/F5H channels are considered. Only Configuration 16 lacks both channels. Other configurations represent different combinations of the absence and presence of channels.

**Figure S2. Topological configurations that are best compatible with all available experimental data.** Models with any of these topologies, which in addition account for product inhibition and competitive inhibition for CCR1, are able to reproduce all available experimental results. Note that at least one channel is present in all configurations.


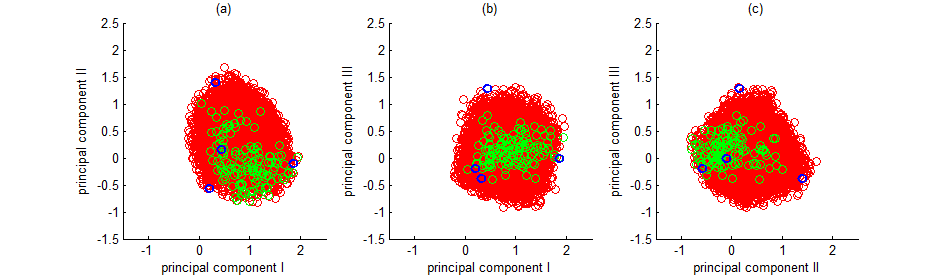


**Figure S3. Parameter distribution along the principal components of the parameter space.** From the initial random sampling in the original parameter space, simulations led to only a limited number of points that successfully satisfied the model criteria. Using PCA, principal directions of the admissible points were identified and used to resample the space (red points). A second round of simulations filtered the randomly generated points again to insure that the model criteria were satisfied. The result is a set of fully admissible parameter values (green point).

**
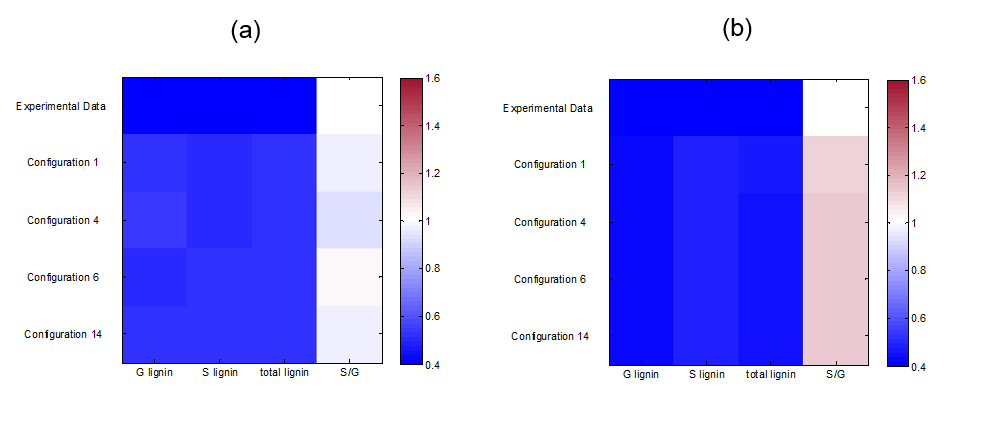
**

**Figure S4. Fold changes in lignin monomer concentrations in PvMYB4 transgenic plants.** The top row represents the average of experimental data normalized with respect to the average of the control plants. Other rows represent the perturbed for PvMYB4 model results normalized with respect to wild type model results in compatible topological configurations. Wild type is set to white in the color bar. H lignin only counts for 3% of total lignin and is not shown in here. (a) The same common parameters are used for all configurations. (b) Best results, where each configuration is simulated using a separate, optimized parameter set.


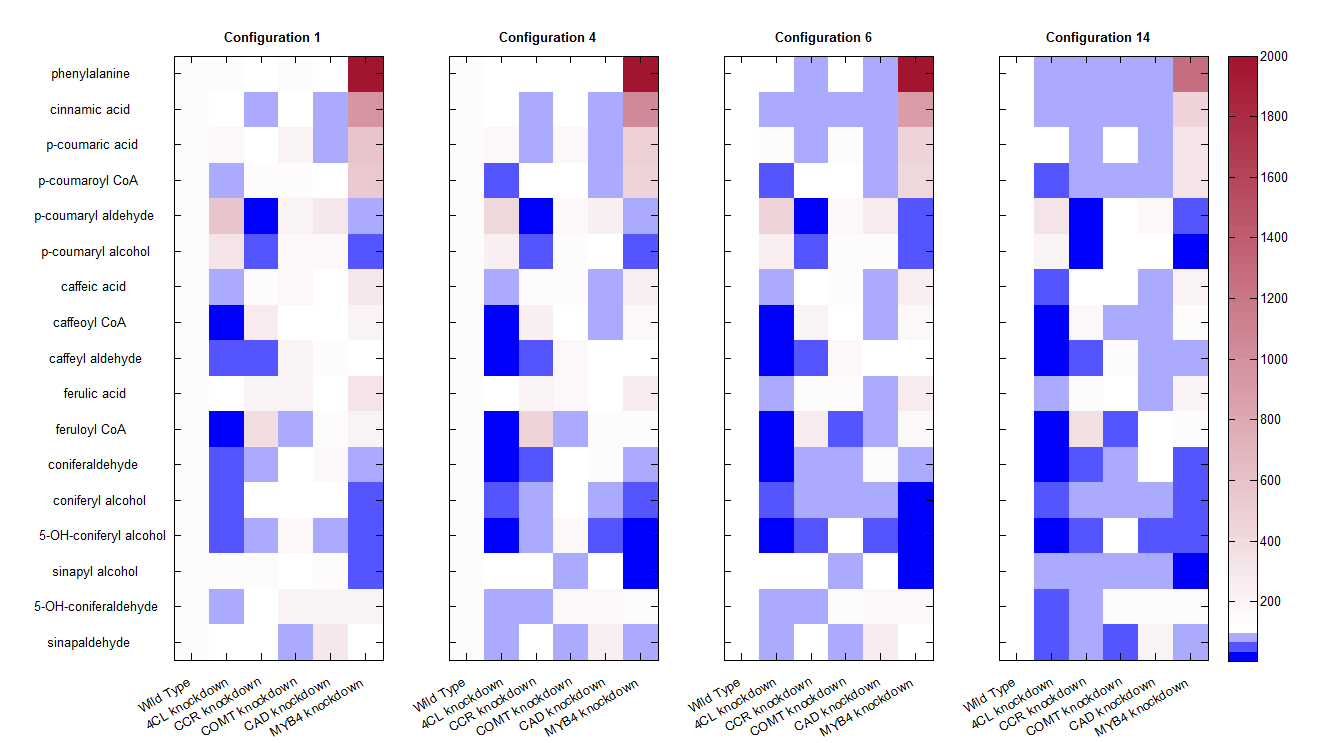


**Figure S5.** Predicted steady-state profiles of key pathway metabolites in the wild type and in single knockdowns and PvMYB4 overexpression as predicted by the model. Concentrations are normalized and the base value is set to 100, which corresponds to white in the color bar. Any increases with respect to the wild type steady state are reflected in the red spectrum and any decreases in the blue spectrum. It is quite evident that the specific model configuration has no significant effect on the predictions.


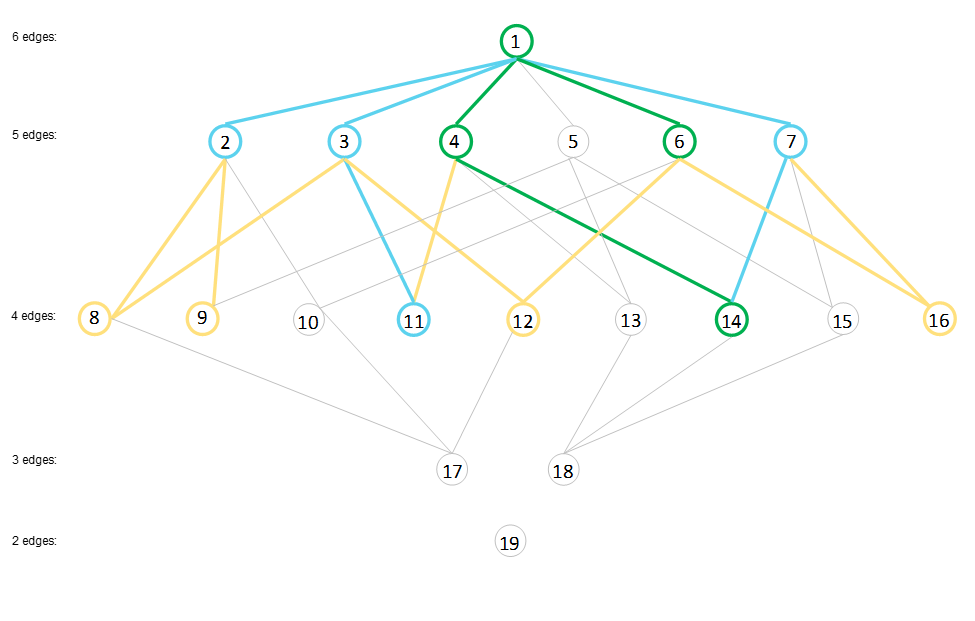


**Figure S6. Map of connectedness of admissible topological configurations.** Two configurations are connected if they differ in only one edge. The best compatible configurations (1, 4, 6, 14; highlighted in green) are connected. The second best compatible configurations (2, 3, 7, 11; highlighted in blue) are also connected. The rest of the compatible configurations (8, 9, 12, 16; highlighted in orange) represent the lease abundant configurations in the solution space. The compatible configurations form a rather tight cluster.

**
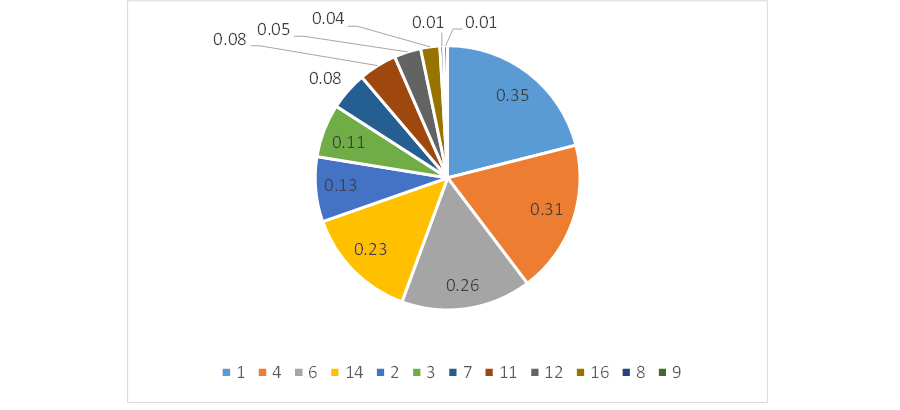
**

**Figure S7. Compatibility ratio in different topological configurations.** Compatibility ratio for each configuration is defined as the number of parameter sets working with this configuration divided by the number of the parameter sets working for all the configurations collectively.

**References**

1. Zhou R, Jackson L, Shadle G, Nakashima J, Temple S, Chen F, Dixon RA: **Distinct cinnamoyl CoA reductases involved in parallel routes to lignin in Medicago truncatula.** *Proceedings of the National Academy of Sciences of the United States of America* 2010, **107:**17803-17808.
