# Supplementary material for: Computational inference of the structure and regulation of the lignin pathway in Panicum virgatum
Source: Biotechnol Biofuels. 2015 Sep 17;8:151. doi: 10.1186/s13068-015-0334-8 (PMC4574612; doi:10.1186/s13068-015-0334-8)
Supplement: Supplementary file 2 — Additional file 2. Principal component analysis. [file 13068_2015_334_MOESM2_ESM.docx]

**Additional file 2**

**Computational Inference of the Structure and Regulation of the**

**Lignin Pathway in *Panicum virgatum***

Mojdeh Faraji, Luis L. Fonseca, Luis Escamilla-Treviño, Richard A. Dixon, Eberhard O. Voit

**Text S2. Principal Component Analysis**

In order to characterize the parameter space of the system, we performed principal component analysis (PCA) on the parameter sets that had been filtered by the model criteria (Figure S1). Principal Components 1 through 4 collectively account for 88% of the variance. The blue circles show the parameters from the original set of simulations, while the red circles represent the set of parameters that was secondarily generated from the blue points using PCA. Finally, the green area is a subset of the red area; it contains the parameter values that successfully passed all model criteria. The straight edges of the red and green areas are due to the imposed biological constraints on the parameter values. Note that the generated area is not a convex hull, that is, the smallest space containing all possible linear combinations of the blue points, but a multi-dimensional “hyperdomain” expanded along the principal directions. Each edge of this hyperdomain is elongated by 20% of the original length in order to cover the principal direction up to 10% off the original blue point.


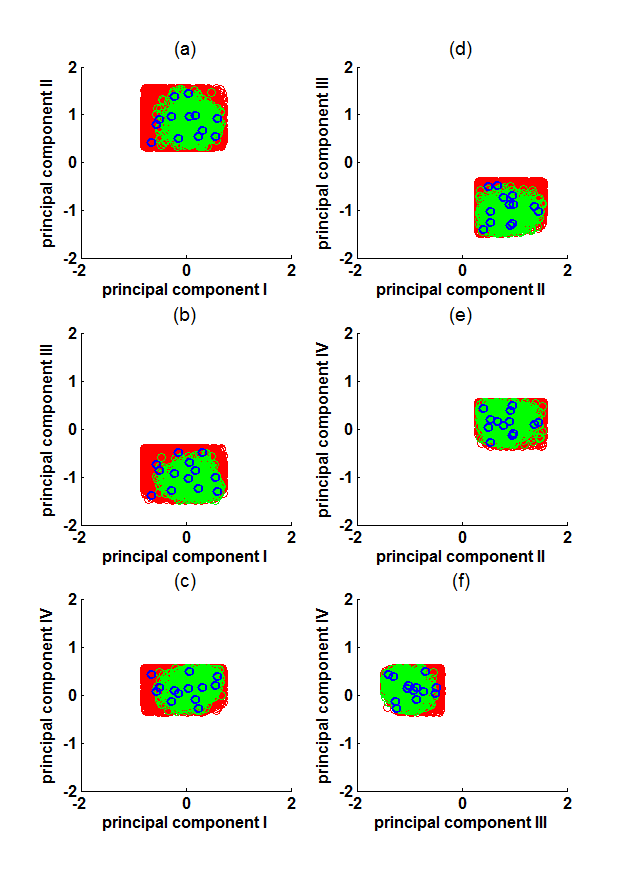


**Figure S8. Parameter distribution along the three principal components of the parameter space.** From the initial sampling in the original parameter space, simulations led to only 13 points that successfully satisfied all model criteria (blue circles). Using PCA, principal directions of the admissible points were identified and used to resample the space with higher density (red points). A second round of simulations filtered the generated points again to insure that all model criteria were satisfied. The result is a set of fully admissible parameter values (green point). Note that the admissible parameter ranges are quite small.
